# Effectiveness of oral health education versus nicotine replacement therapy for tobacco cessation- a parallel randomized clinical trial

**DOI:** 10.4317/jced.52738

**Published:** 2016-02-01

**Authors:** Mitali Raja, Sabyasachi Saha, Vamsi Krishna-Reddy, Shafaat Mohd, Ridhi Narang, Poonam Sood

**Affiliations:** 1 Senior Lecturer, Department of Public Health Dentistry, Rama Dental College Hospital and Research Centre, Kanpur, Uttar Pradesh; 2 Professor and Head, Department of Public Health Dentistry, Sardar Patel Postgraduate Institute of Dental and Medical Sciences, Lucknow, Uttar Pradesh; 3 Reader, Department of Public Health Dentistry, Sardar Patel Postgraduate Institute of Dental and Medical Sciences, Lucknow, Uttar Pradesh; 4 Senior Resident, Department of Public Health Dentistry, Post Graduate Institute of Dental Sciences, Rohtak, Haryana; 5 Assistant Professor, Department of Public Health Dentistry, Dr Harvansh Singh Institute of Dental Sciences and Hospital, Chandigarh

## Abstract

**Background:**

India has millions of tobacco users. It is the leading cause of deaths due to oral cancer and hence needs effective strategies to curb it. Hence the aim of present study was to evaluate and compare the effectiveness of Oral Health Education (OHE) and Nicotine Replacement Therapy (NRT) in tobacco cessation.

**Material and Methods:**

The clinical trial consisted of Manohar Lal Kapoor (MLK) factory workers (n= 40) giving history of tobacco consumption (smoking/smokeless) within past 30 days. They were randomized into OHE (n=20) and NRT (n=20) groups. Baseline evaluation (demographic, smoking/ smokeless behaviour) was done. Fagerstrom test was used for Nicotine Dependence (FTND) and to assess nicotine addiction level. Follow up was done at an interval of 1week, 2 weeks, 1 month, 2 months and 3 months to assess the reduction in the mean FTND score. “Nano-CheckTM Rapid Nicotine test” was used for the qualitative detection of cotinine in human urine. Appropriate statistical analysis was performed (Paired and Unpaired t test).

**Results:**

In both OHE and NRT group there was a significant reduction (*p*< 0.00001) in mean Fagerstrom score at every follow up but when both the groups were compared mean Fagerstrom score reduction was more in NRT than OHE at all time interval though it was not statistically significant (*p*>0.05).

**Conclusions:**

NRT is better than OHE when both the groups were compared. However, it was found that any intervention given to tobacco users either NRT or OHE is helpful for the patients in the process of quitting tobacco.

** Key words:**Tobacco cessation, nicotine replacement therapy, oral health education, fagerstrom test, urine cotinine.

## Introduction

Tobacco is a well-acknowledged social and health evil. The history of tobacco use traces back to the dawn of human civilization and has deeply entrenched into the human society since time immemorial. The social, economic, and health impact of tobacco has been a subject of intense debate over the recent decades (1). It is the greatest disease-producing product, with its prevalent addictive habit influencing the behavior of human beings for more than four centuries (2). It is consumed orally in a variety of forms such as smoking and chewable forms (2). According to WHO (2009) consumption of tobacco has been growing at the rate of 2% to 5% per annum (1). Tobacco is a risk factor for oral cancer, oral cancer recurrence, adult periodontal diseases, and congenital defects such as cleft lip and palate in children whose mother smokes during pregnancy. Tobacco use suppresses the immune system’s response to oral infection, retards healing following oral surgical and accidental wounding, promotes periodontal degeneration in diabetics and adversely affects the cardiovascular system. These risks increase when tobacco is used in combination with alcohol or areca nut. Most oral consequences of tobacco use impair quality of life be they as simple as halitosis or as complex as oral birth defects, as common as periodontal disease or as troublesome as complications during healing (3).

Globally, the total number of tobacco-attributable deaths from ischaemic heart disease, lung cancer and other diseases is projected to rise from 5.4 million in 2004 to 8.3 million in 2030 (4,5). It is a serious public health challenge in several regions of the world. It has assumed the dimension of an epidemic resulting in enormous disability, disease and death (2). It is a multi-dimensional addiction that causes psychological, physiological and behavioural dependence on nicotine (6).

Scenario is no different in India rather worse. We have 16.2% current smokers and 20.5% tobacco chewers. Beedi is the most popular form of tobacco smoking, followed by cigarette smoking. Pan with tobacco is the major chewing form of tobacco (7). A recent nationwide study on smoking and mortality in India estimated that cigarette and beedi smoking causes about 5% of all deaths in women and 20% of all deaths in men aged 30-69 years, totalling to 1 million deaths per year in India (1).

Controlling and preventing the further use of tobacco is utmost important. Nicotine dependence and the degree of that dependence is determined by individual and psychosocial factors as well as combinations of these factors (8). India has played a leadership role in global tobacco control. With the growing evidence of harmful and hazardous effects of tobacco, the Government of India enacted various legislations and comprehensive tobacco control measures (9). These include advertising bans, package labelling, prohibition of smoking at public places and raising taxes. Such policies encourage the social norm of non-smoking and increase the demand for cessation services (10).

Oral health education and nicotine replacement therapy are also gaining popularity. In fact, tobacco cessation efforts in India began in the context of primary, community based interventions for cancer control in the 1980s and 1990s (10). In 2002, 13 Tobacco Cessation Clinics (TCCs) were set up to provide the first formal cessation intervention in India. It is hypothesised that oral health education and nicotine replacement therapy are effective for tobacco cessation. Few studies across the world have proved that NRT and OHE are effective tools in motivating patients to quit tobacco (11-14). However none have compared which technique can be best used amongst two or should it be the combination and permutation of these techniques. Hence present study was conducted to compare the effectiveness of Oral Health Education and Nicotine Replacement Therapy for tobacco cessation.

## Material and Methods

-Study Design

A parallel randomized clinical trial was designed to evaluate and compare the effectiveness of oral health education and nicotine replacement therapy among tobacco users of MLK factory, Lucknow. Ethical clearance was taken from Institutional Ethical Committee of Sardar Patel Postgraduate Institute of Dental and Medical Sciences Lucknow. Permission was also obtained from MLK factory owner. Investigator was trained and calibrated in the department of Public Health Dentistry in Sardar Patel Postgraduate Institute of Dental and Medical Sciences Lucknow to conduct study.

-Study participants

The study was conducted between June 2013 and September 2013 in the MLK factory. MLK factory has been adopted by Tobacco Cessation Centre of Public Health Dentistry Department of Sardar Patel Postgraduate Institute of Dental and Medical Sciences, Lucknow. Inclusion criteria consisted of: workers who were permanent residents of that area and reachable by phone, gave informed consent and had history of tobacco (smoking/smokeless) consumption within past 30 days. Those enrolled in another cessation program, using pharmacotherapy for cessation, pregnant/breastfeeding, or diagnosed with an acute cardiac or respiratory problem were excluded.

-Procedure 

Based on inclusion and exclusion criteria 40 subjects were enrolled in the study. Written informed consent was obtained and baseline evaluation (on demographics, smoking/smokeless behaviour) done. The patients were randomly assigned to either Oral Health Education (OHE; Group I) or Nicotine Replacement Therapy (NRT; Group II) using lottery system. Demographic information included age, area of residence, education, marital status, frequency of tobacco consumption per day and duration of regular tobacco use (in years). Fagerstrom test for Nicotine Dependence (FTND) was used to assess factory workers nicotine addiction level (11,12). The Fagerström Test for Nicotine Dependence is a standard instrument for assessing the intensity of physical addiction to nicotine. The test was designed to provide an ordinal measure of nicotine dependence related to cigarette smoking. It contains six items that evaluate the quantity of cigarette consumption, the compulsion to use, and dependence.

In scoring the Fagerstrom Test for Nicotine Dependence, yes/no items are scored from 0 to 1 and multiple-choice items are scored from 0 to 3. The items are summed to yield a total score of 0-10. The higher the total Fagerström score, the more intense is the patient’s physical dependence on nicotine (11,12). Follow up was done in both the groups at an interval of 1week, 2 weeks, 1 month, 2 months and 3 months to assess the reduction in the mean FTND score. At the end “Nano-CheckTM Rapid Nicotine test” was used for the qualitative detection of cotinine in urine.

-Nicotine Replacement Therapy (NRT): Prior information regarding the use of Nicotine Replacement therapy chewing gum and its other effects were explained to the patients. They were told that Nicotine Replacement therapy supply low doses of nicotine and do not contain the toxins found in smoke. The goal of therapy was to cut down cravings for nicotine and ease the symptoms of nicotine withdrawal. Two mg Nicotine Replacement gums were given to the workers depending upon the frequency of tobacco intake. They were asked to chew the gum until they perceived a peppery or tingling sensation, thereafter instructed to place the gum in their cheek region and slowly chew it again. When the peppery taste or tingle was almost gone (in about a minute), they were again asked to start chewing the gum piece slowly. They were instructed to stop chewing when the taste or tingle returns and shift the gum to a different place in their mouth every time. It was advised to repeat the chewing and shifting process of the gum till most of the nicotine is gone. Any citrus fruits/juices, or any other beverages, soft drinks were to be avoided 1 hour before NRT. Dose adjustment was done accordingly.

-Oral Health Education (OHE): The intervention served as a control arm but provided information on the harmful effects of tobacco use. It included initiating assessment and intervention using the 5 A’s which comprised of Asking about the tobacco use, Advising the patient to quit, Assessing the patient’s willingness to quit, Assisting the patient to quit by counselling using a pictorial depiction of the various harmful side effects of tobacco in the form of a power point presentation and booklet specifically designed for this study and arranging follow up contacts for relapse prevention by psychological support. Patients were given education that tobacco can increase risk for periodontal (gum) disease-a leading cause of tooth loss and sensitivity, delayed healing after a tooth extraction or other oral surgery, oral cancer, bad breath, stained teeth and tongue, diminished sense of taste and smell. The sessions lasted from 45-60 minutes. All these motivational messages were repeated at every follow up (i.e at 1week, 2 weeks, 1 month, 2 months and 3 months). At the last follow up that is at 3 months, Nano- CheckTM Rapid Nicotine test was used to detect cotinine in human urine.

-Statistical analysis 

SPSS version 20 was used to analyze the data. Chi square test, paired and unpaired‘t’ test were used for intra-group and inter-group comparisons. *P*- value < 0.05 was considered statistically significant.

## Results

The study evaluated and compared effectiveness of OHE and NRT as tobacco cessation intervention on MLK factory workers. All the subjects were males, mostly were from rural areas (70% in OHE group nd 75% in NRT group) and cleared middle school. Majority consumed chaini kahaini (40% in OHE group and 35% in NRT group) and panmasala (60% in OHE and NRT group respectively) daily for an average of 5 years ( Table 1).

**Table 1:**
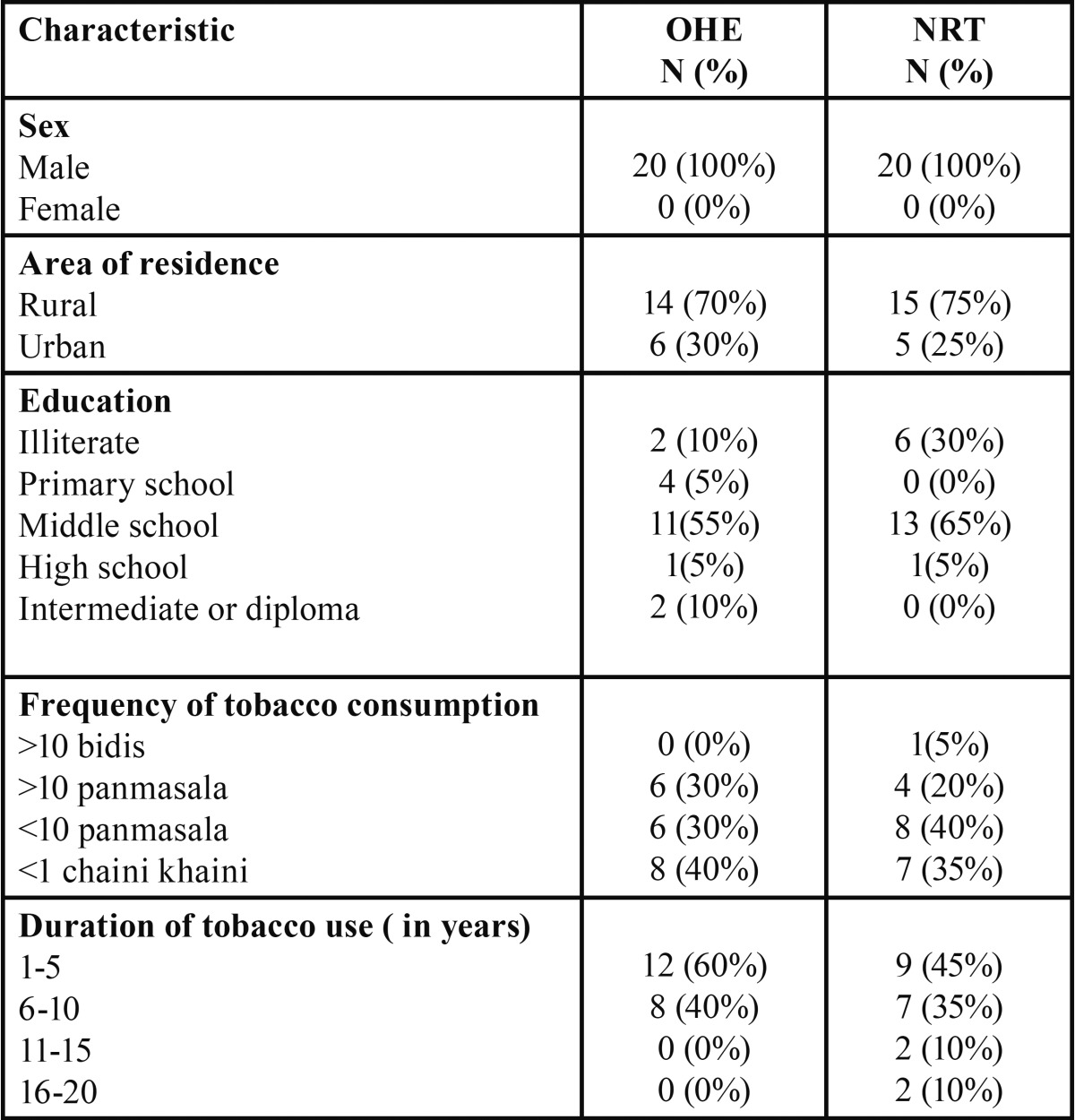
Distribution of study subjects.

 Table 2 shows that at baseline the mean FTND Score was 6.70, at 1st week it was reduced to 5.65, at 2nd week (4.60), at 1st month (3.75), at 2nd month (2.90), at 3rd month (2.25) and that there was a significant reduction in mean FTND score from baseline (6.70) to 3rd month (2.25) in Oral health education group (OHE).

**Table 2:**
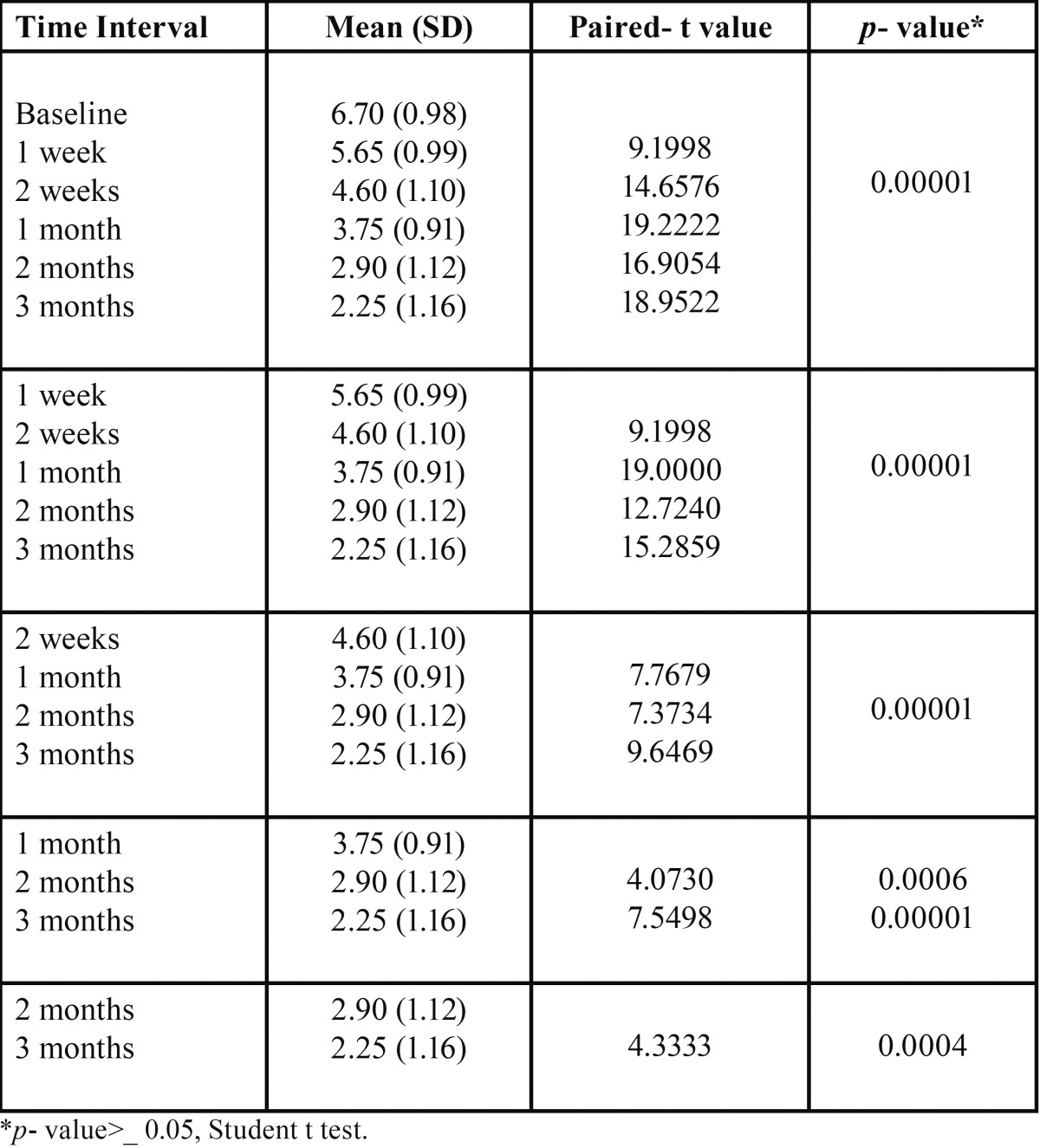
Comparison of Fagerstorm scores within OHE group.

 Table 3 shows that at baseline the mean FTND Score was 6.85, at 1st week it was reduced to 5.45, at 2nd week (4.45), at 1st month (3.50), at 2nd month (2.15), at 3rd month (1.65) and that there was a significant reduction in mean FTND score from baseline (6.85) to 3rd month (1.65) in Nicotine replacement group (NRT).

**Table 3:**
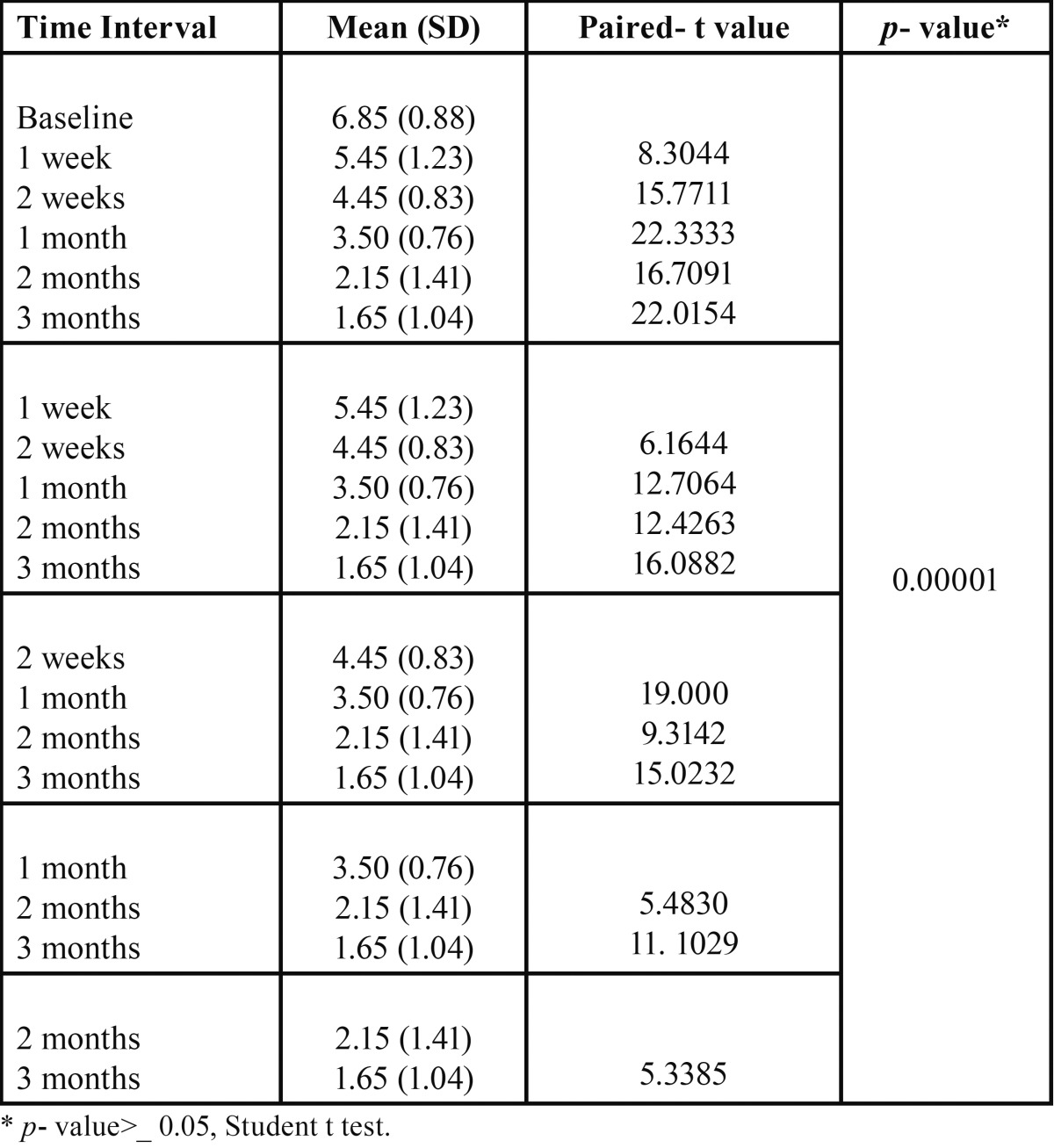
Comparison of Fagerstorm scores within NRT group.

**Figure 1:**
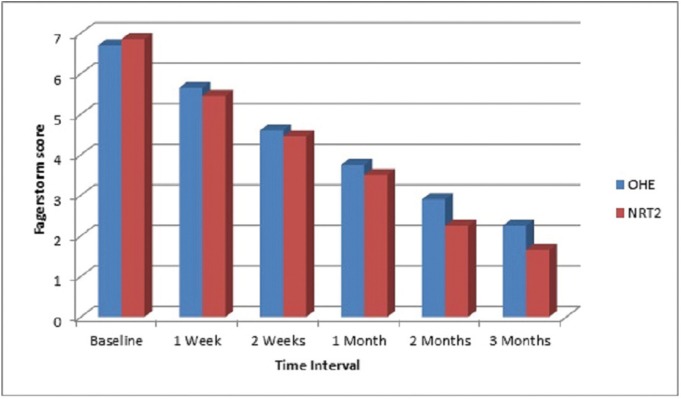
Comparison of Fagerstorm scores between OHE & NRT groups at different time intervals.

 Table 4 and figure 1 compares reduction of mean FTND score in both Oral health education and Nicotine replacement therapy group at different intervals. At baseline the mean FTND score was 6.70 in OHE group and 6.85 in NRT group which to 2.25 in OHE group and 1.65 in NRT group at 3rd month. Mean score reduction was more in nicotine replacement therapy group (NRT) than oral health education group (OHE) at all time interval but it was not statistically significant. Nano-CheckTM Rapid Nicotine test was used to detect cotinine in human urine. The result was negative (two colored lines appeared) in 5 factory workers in NRT group and 3 factory workers in OHE group. Thus it was found that 5 workers from the NRT group and 3 workers from OHE group successfully quitted tobacco.

**Table 4:**
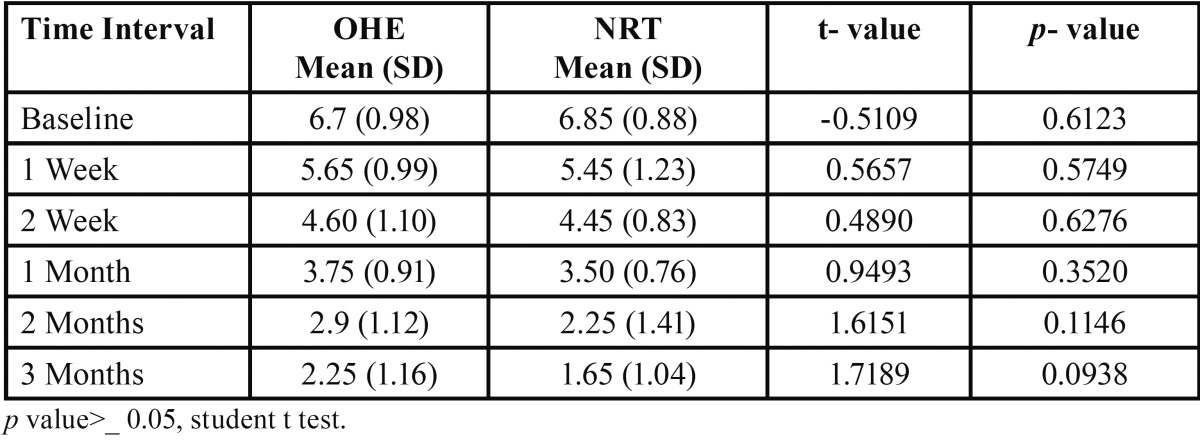
Comparison of Fagerstorm scores between OHE & NRT groups at different time intervals.

## Discussion

The study was done to assess and compare the effectiveness of nicotine replacement therapy (NRT) and oral health education (OHE). Both NRT and OHE performed well but when both the groups were compared mean score reduction was more in NRT than OHE. However, it was not statistically significant (*p*>0.05). This suggests that the differences in the interventions are due to different strategies adopted. It may also depend on expertise of the clinician employing these strategies and patient profile, attitude, belief and response.

Nicotine replacement therapy (NRT) is the most commonly used intervention for smoking cessation introduced almost 20 years back. It was designed to replace blood nicotine levels, minimising withdrawal symptoms like depression, anxiety, weight gain, insomnia, irritability etc (17,18). It is considered safe as it is devoid of all the carcinogens and harmful chemicals contained in a cigarette or beedi (19). OHE was also used as interventional strategy in few studies. Counselling patients can be one of the forms of imparting knowledge and motivating patients to quit smoking.

Direct comparisons are not possible with other studies as none have compared two interventions together in a way done in the present study. However few studies have compared two strategies with placebo independently. Studies have shown that showed that brief physician assistance, along with nicotine replacement therapy, could help well-motivated smokers to quit (11,13).

Etter *et al.* reported that NRT was only slightly more effective than placebo even in heavy smokers (19). Hand *et al.* showed that not much significant difference is seen if patients are only on NRT or on combination of NRT and advice (12). In a Cochrane review of 35,600 participants it was found that NRT was more effective than placebo or no treatment given (20). A study conducted by Pai A and Prasad S noticed that patients with very low or low dependence had good response in the placebo group (68% and 47.6% respectively) (14). In the counseling group maximum response was seen in the medium dependence patients followed by the very low group (61% and 59% respectively). In NRT group maximum response was seen in very high dependence patients (78.7%). Thus NRT performed better than OHE, when both the groups were compared mean score reduction was more in NRT then OHE. A study conducted by Cornuz *et al.* showed that smokers who do not intend to quit smoking, physicians should inform and sensitise them about tobacco use and cessation (21). For smokers who are dissonant, physicians should use motivational strategies, such as discussing barriers to cessation and their solutions. For smokers ready to quit, the physician should show strong support, help set a quit date, prescribe pharmaceutical therapies for nicotine dependence, such as nicotine replacement therapy.

There were two patients in the present study who reported quitting tobacco but after conducting Nano-CheckTM Rapid Nicotine test they were found positive. The cotinine present in their urine sample, showed that they falsely reported that they had quit tobacco. Verbal statements made by the patients need to be supported by appropriate laboratory tests. Also relapse for a shorter duration may have happened which could have led to the positive results.

Both the findings of previous studies as well as the findings of the present study indicate the need for further investigation Future research should include studies with larger samples of tobacco users. A through awareness is the key to make people realize health related hazards and to increase the willingness to quit the habit. The goal of any intervention must be complete long term abstinence from the habit as the true objective is to decrease or eliminate smoking induced morbidity and mortality. NRT should be given with brief counselling as a routine therapy to all tobacco users who indicate that they are prepared to try to stop the habit. OHE can in general raise the awareness and motivate patients to think about quitting. Though Nicotine replacement therapy performed better than oral health education, however it was not found to be statistically significant. Hence to conclude, any intervention either NRT or OHE given to tobacco users was helpful to the patients in quitting the habit of tobacco and both are effective for tobacco cessation.

However the results should be generalized carefully, as the present study was done among factory workers. Their habits, socio economic status etc might be different from the general population. Further studies with larger sample size are recommended.

## References

[1] Rao V, Chaturvedi P (2010). Tobacco and health in India. Indian Journal of Cancer.

[2] Tiwari RV, Megalamanegowdru J, Gupta A, Agrawal A, Parakh A, Pagaria S (2014). Knowledge, attitude and practice of tobacco use and its impact on oral health status of 12 and 15 year-old school children of Chhattisgarh, India. Asian Pac J Cancer Prev.

[3] Petersen PE (2003). Tobacco and Oral Health – the Role of the World Health Organization. Oral Health & Prev Dent.

[4] Danaei G, Ding EL, Mozaffarian D, Taylor B, Rehm J, Murray CJL (2009). The Preventable causes of death in the United States: comparative risk assessment of dietary, lifestyle, and metabolic risk factors. PLoS Med.

[5] Inoue T (2004). Cigarette smoking as a risk factor of coronary artery disease and its effects on platelet function. Tob Induc Dis.

[6] Mokdad AH, Marks JS, Stroup DF, Gerberding JD (2004). Actual causes of death in the United States, 2000. JAMA.

[7] Kalyanpur R, Pushpanjali K, Prasad KVV, Chhabra KG (2012). Tobacco cessation in India: A contemporary issue in public health Dentistry. Indian J Dent Res.

[8] Schmidt A, Neumann M, Wirtz M, Ernstmann N, Staratschek-Jox A, Stoelben E (2010). The influence of occupational stress factors on the nicotine dependence: a cross sectional study. Tob Induc Dis.

[9] Kaur J, Jain DC (2011). Tobacco Control Policies in India: Implementation and Challenges. Indian J Public Health.

[10] Kaur P, Thomas DR, Govindasamy E, Murhekar MV (2014). Monitoring smoke-free laws in restaurants and educational institutions in Chennai, India. Natl Med J India.

[11] Reid RD, Pipe A, Dafoe WA (1999). Is telephone counselling a useful addition to physician advice and nicotine replacement therapy in helping patients to stop smoking? A randomized controlled trial. CMAJ.

[12] Hand S, Edwards S, Campbell IA, Cannings R (2002). Controlled trial of three weeks nicotine replacement treatment in hospital patients also given advice and support. Thorax.

[13] Molyneux A, Lewis S, Leivers U, Anderton A, Antoniak M, Brackenridge A (2003). Clinical trial comparing nicotine replacement therapy (NRT) plus brief counselling, brief counselling alone, and minimal intervention on smoking cessation in hospital inpatients. Thorax.

[14] Pai A, Prasad S (2012). Attempting Tobacco Cessation - An Oral Physician's Perspective. Asian Pac J Cancer Prev.

[15] Ebbert JO, Patten CA, Schroeder DR (2006). The Fagerstrom Test for Nicotine Dependence-Smokeless Tobacco (FTND-ST). Addict Behav.

[16] Heatherton TF, Kozlowski LT, Frecker RC, Fagerstrom KL (1991). The Fagerstrom Test for Nicotine Dependence: a revision of the Fagerstrom Tolerance Questionnaire. Bri J Addict.

[17] Cahall EJ (2004). Assisting with tobacco cessation. J Vascular Nurs.

[18] Cepeda BA, Reynoso JT, Erath S (2004). Meta-analysis of the efficacy of nicotine replacement therapy for smoking cessation: differences between men and women. J Consulting and Clin Psycho.

[19] Dar R, Etter JF, Stronguin F (2005). Assigned versus perceived placebo effects in nicotine replacement therapy for smoking reduction in swiss smokers. J Consulting and Clin Psycho.

[20] Kumar R, Prakash S, Kushwah AS, Vijayan VK (2010). Breath carbon monoxide concentration in cigarette and bidi smokers in India. Indian J Chest Dis Allied Sci.

[21] Cornuz J (2007). Smoking cessation interventions in clinical practice. Eur J Vasc Endovasc Surg.

